# Experiences of the Data Monitoring Committee for the RECOVERY trial, a large-scale adaptive platform randomised trial of treatments for patients hospitalised with COVID-19

**DOI:** 10.1186/s13063-022-06824-6

**Published:** 2022-10-18

**Authors:** Peter A. G. Sandercock, Janet Darbyshire, David DeMets, Robert Fowler, David G. Lalloo, Mohammed Munavvar, Natalie Staplin, Adilia Warris, Janet Wittes, Jonathan R. Emberson

**Affiliations:** 1 Centre for Clinical Brain Sciences, University of Edinburgh, Edinburgh, UK; 2 University College London, London, UK; 3 School of Medicine and Public Health, University of Wisconsin-Madison, Madison, USA; 4 Department of Medicine and Interdepartmental Division of Critical Care Medicine, University of Toronto, Toronto, Canada; 5 Liverpool School of Tropical Medicine, Liverpool, UK; 6 Lancashire Teaching Hospitals and University of Central Lancashire, Preston, UK; 7 Medical Research Council Population Health Research Unit, Clinical Trial Service Unit and Epidemiological Studies Unit, Nuffield Department of Population Health, University of Oxford, Oxford, UK; 8 MRC Centre for Medical Mycology, University of Exeter, Exeter, UK; 9 Wittes LLC, Washington D.C., USA

## Abstract

**Aim:**

To inform the oversight of future clinical trials during a pandemic, we summarise the experiences of the Data Monitoring Committee (DMC) for the Randomised Evaluation of COVID therapy trial (RECOVERY), a large-scale randomised adaptive platform clinical trial of treatments for hospitalised patients with COVID-19.

**Methods and findings:**

During the first 24 months of the trial (March 2020 to February 2022), the DMC oversaw accumulating data for 14 treatments in adults (plus 10 in children) involving > 45,000 randomised patients. Five trial aspects key for the DMC in performing its role were: a large committee of members, including some with extensive DMC experience and others who had broad clinical expertise; clear strategic planning, communication, and responsiveness by the trial principal investigators; data collection and analysis systems able to cope with phases of very rapid recruitment and link to electronic health records; an ability to work constructively with regulators (and other DMCs) to address emerging concerns without the need to release unblinded mortality results; and the use of videoconferencing systems that enabled national and international members to meet at short notice and from home during the pandemic when physical meetings were impossible. Challenges included that the first four treatments introduced were effectively ‘competing’ for patients (increasing pressure to make rapid decisions on each one); balancing the global health imperative to report on findings with the need to maintain confidentiality until the results were sufficiently certain to appropriately inform treatment decisions; and reliably assessing safety, especially for newer agents introduced after the initial wave and in the small numbers of pregnant women and children included. We present a series of case vignettes to illustrate some of the issues and the DMC decision-making related to hydroxychloroquine, dexamethasone, casirivimab + imdevimab, and tocilizumab.

**Conclusions:**

RECOVERY’s streamlined adaptive platform design, linked to hospital-level and population-level health data, enabled the rapid and reliable assessment of multiple treatments for hospitalised patients with COVID-19. The later introduction of factorial assessments increased the trial’s efficiency, without compromising the DMC’s ability to assess safety and efficacy. Requests for the release of unblinded primary outcome data to regulators at points when data were not mature required significant efforts in communication with the regulators by the DMC to avoid inappropriate early trial termination.

**Supplementary Information:**

The online version contains supplementary material available at 10.1186/s13063-022-06824-6.

## Introduction

A Data Monitoring Committee (DMC) for a randomised trial is a group of individuals, independent of the trial investigators, who are responsible for ensuring the safety of both the patients in the trial as well as the safety of future patients whose treatment may be influenced by the results of the trial [1, 2]. The DMC performs this role by periodically reviewing interim, unblinded data and then making recommendations to the trial investigators on whether the trial should continue as planned or stop early because of evidence of benefit or hazard. Oversight provided by an independent DMC is particularly important for trials conducted during public health emergencies, such as the HIV/AIDS epidemic or the COVID-19 pandemic [3]. However, the effective operation of a DMC in this setting also brings some specific challenges. The RECOVERY trial was (and, at the time of writing, still is) a large-scale randomised adaptive platform trial evaluating widely applicable treatments among patients admitted to hospital with COVID-19 (see http://www.recoverytrial.net/). The current paper, written by the members of the RECOVERY DMC, describes some of our work that was somewhat outside the ‘run of the mill’ experience of typical DMCs: we hope these examples may inform DMC operations of future trials and the approach taken by those designing, conducting, and regulating clinical trials. Although these examples all relate to the COVID-19 pandemic, the experiences and lessons learned also have relevance to trials more generally.

## Methods

During the first 2 years of the trial (March 2020 to February 2022), RECOVERY evaluated 14 different treatments in adults, leading to 10 publications to date [4–13]. Below, we summarise the decisions around four of those treatments (hydroxychloroquine, dexamethasone, casirivimab + imdevimab, and tocilizumab) (further information on these and the other treatments assessed are provided in the Additional file 1). We begin, however, by describing some aspects of the trial which were key to its success or which posed particular challenges to the DMC. Tables 1 and 2 summarise these challenges as well as some lessons for future DMCs and the approach taken by those designing, conducting, and regulatory clinical trials in pandemics.

**Table 1:** Questions. Difficult issues frequently discussed at DMC meetings

• How long should patients continue to be randomised to a treatment when the early data suggests a marginally negative trend, especially if the treatment is widely promoted as effective?
• How important is it that a negative result is ‘convincingly’ neutral, particularly when it is competing against other potentially beneficial treatments or when there are other treatments that could be introduced in its place?
• When is it appropriate for the DMC to perform their own futility analyses when such analyses have not been specified in the DMC charter?
• How to react to observational studies that are swaying opinions about a treatment?
• How much belief to put in the results of multiple interim subgroup analyses?
• How to respond to regulators’ requests for unblinded interim results?
• How to interact with other DMCs and what information should be shared with them?

**Table 2:** Lessons for DMC and trialists in future pandemic (and non-pandemic) trials

• Emphasise the need for wider trust in the judgement of a properly convened and expert Independent DMC (with names of DMC members available publicly to facilitate this trust)
• Liaise with regulators at an early stage to ensure that the role of the DMC is properly understood in order to avoid later requests for unblinded interim results
• Employ factorial designs where appropriate to allow simultaneous unbiased assessments of multiple treatments as rapidly as possible
• Ensure DMC includes members with a wide range of expertise in clinical trials, statistics, epidemiology, and clinical medicine and include individuals with expertise in monitoring the safety and efficacy of treatments in any special populations (e.g. children and pregnant women)
• DMC should adhere to the principle that decisions are made by the chief investigators and steering committee unless there are either safety concerns or a definitive efficacy result
• Ideally enrol a second unblinded statistician to produce reports for the DMC to cross-check results and provide resilience in case of illness

### Rapid trial set-up and initiation of a DMC with appropriate expertise

**Fig. 1:**
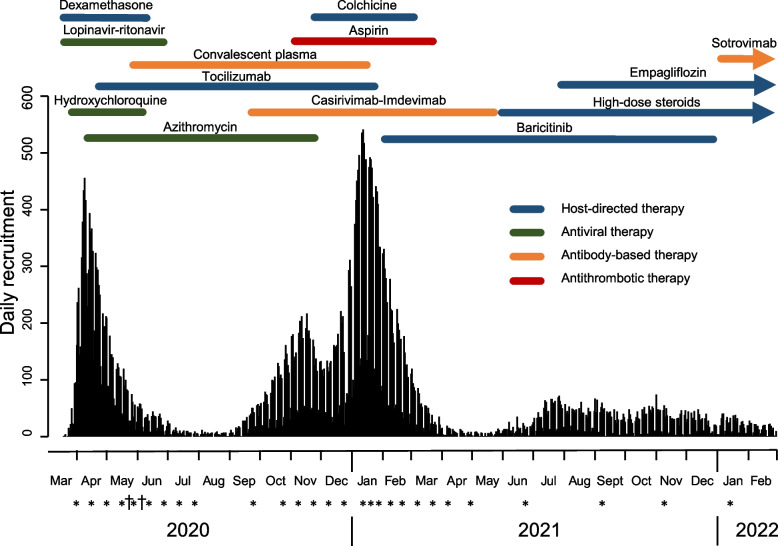
Daily recruitment into the RECOVERY trial and periods of each main treatment assessment, March 2020 to February 2022. Scheduled (*) and emergency (†) DMC meetings. In addition to the main assessments shown, dimethyl fumarate vs usual care was assessed in 713 adults between March and November 2021, while additional assessments in children with paediatric multisystem inflammatory syndrome temporally associated with COVID-19 (PIMS-TS) have involved intravenous immunoglobulin vs high-dose methylprednisolone vs usual care (August 2020 to July 2021, 141 children) and anakinra vs tocilizumab vs usual care (May 2020 to January 2022, 75 children)

In view of the impending arrival of the COVID-19 pandemic in the UK in early 2020, the trial was approved and the DMC convened in a remarkably short time. The trial principal investigators sought to establish the trial quickly enough that recruitment was open by the time the number of patients admitted to hospitals in the UK with COVID started to rise. The investigators began to draft the protocol on 9 March 2020, submitted it for ethics and regulatory approval on 13 March, and randomised the first patient on 19 March. The trial protocol is available at www.recoverytrial.net. The daily rates of recruitment over the next 24 months are shown in Fig. 1. On 23 March 2020, the UK government announced a national lockdown. The DMC first met on 31 March, by which time 151 patients from 30 UK hospitals had been recruited. The members of the DMC were nominated by the trial chief investigators and collectively included people with substantial expertise and experience with DMC work and large-scale clinical trials as well as physicians with relevant clinical expertise [14]. DMCs vary in size but often include six or fewer individuals. For RECOVERY, however, a slightly larger DMC was selected to cover the range of expertise needed, deal with the likely complexity of the work, and provide some resilience in case members of the DMC themselves became unwell with COVID-19 (or other illnesses); to date, no patients or patient advocates have been included on the DMC. This experience and expertise proved invaluable from the outset. Two new members joined in 2022 to further broaden the diversity of medical expertise. All members declared any potential conflicts of interest. The use, from the outset, of videoconferencing systems enabled meetings to be arranged at short notice, easy participation by national and international members, and the DMC to conduct the open and closed sessions efficiently, while working from home during the pandemic. All meetings were conducted online. The University of Oxford (the trial Sponsor) indemnified us against any legal action we may face performing the role outlined in our DMC Charter (Additional file 1). We were not responsible for selecting or advising on new treatments to be assessed and were not paid for our work.

### Clear strategic planning and communication, flexible approach to sample size

Pogue [15] and DeMets [16] have emphasised that the complex decisions a DMC must make depend on clear, concise, and timely communication from the study chief investigators (or Steering Committee) and the data team preparing the DMC reports. In RECOVERY, the investigators provided a clear strategic framework for the trial which was regularly adapted to the changing pandemic. Importantly, in the open session of every DMC meeting, the investigators provided a concise update of trial progress, anticipated number of severe COVID-19 cases, external information from other trials, their plans, and any decisions from the Steering Committee. (The Steering Committee is listed in the trial protocol and includes the trial chief investigators plus other research academics from both within and outside the University of Oxford.) They intended the trial to generate evidence that was reliable enough to change practice worldwide and the initial sample size was simply ‘as many as possible’. The DMC charter (see Additional file 1) prescribed no hard ‘stopping rules’. Instead, it was simply written that, *The DMC will determine if, in their view, the randomised comparisons in the study have provided evidence on mortality that is strong enough (with a range of uncertainty around the results that is narrow enough) to affect national and global treatment strategies* [17]. This wording, as well as all other aspects of the Charter, was approved by the DMC members. The primary outcome was 28-day mortality. For the four interventions included in the initial phase of the trial (lopinavir-ritonavir, dexamethasone, hydroxychloroquine, azithromycin), the investigators and the DMC understood that, with two patients allocated usual care for every one allocated each treatment, each assessment would likely need several thousand patients. (The randomisation of two patients to usual care for every one allocated each treatment ensures that each treatment can be reliably compared against the, largely overlapping, control groups of patients.) By mid-May 2020, it had become clear that the overall 28-day mortality rate was about 25%. The investigators then advised us that 2000 patients allocated to each active arm vs 4000 patients allocated to each active arm’s own control group (i.e., usual care alone) would be needed to give 90% power at two-tailed *p* = 0.01 to detect a proportional reduction of about one-fifth.

### Frequency of DMC meetings

At the first meeting on 31 March 2020, daily recruitment was increasing rapidly (Fig. 1) and was expected to be high for at least several more weeks or even months. Consequently, we agreed with the investigators on an initial schedule of meetings every 2 weeks. We considered the potential impact of multiple interim assessments on the final type I error level used for significance testing by assuming that we would not consider recommending stopping a treatment early for a benefit unless there was *at least* a 3 to 3.5 standard error reduction in mortality (consistent with the language used in the charter that evidence should be strong enough to *affect national and global treatment strategies*) [18, 19]. This revealed that ten or even twenty interim assessments for each treatment would have only a marginal impact on the overall type I error rate. Meetings were therefore held every 2 weeks until the 9th scheduled meeting on 30 July 2020, after which the frequency was reduced in line with the slowing of the UK epidemic in the Summer of 2020. In total, during the first year, we had 22 scheduled DMC meetings and 2 emergency review meetings (both of which were arranged in response to specific requests from the UK Medicines and Healthcare products Regulatory Agency [MHRA]: see below).

### High recruitment rates: value of the scalable routine data linkage systems

In the first few months of the trial, the chief source of outcome data available to us was the follow-up form completed by the doctors and nurses at hospital discharge, death or at 28 days, whichever was sooner. During the first peak (April 2020), recruitment exceeded 400 patients per day while during the second peak (January 2021), it exceeded 500 per day (Fig. 1). As a consequence, at certain times, a lot of follow-up information (particularly for non-fatal outcomes) was incomplete simply because patients had not yet reached 28 days post-randomisation. As the trial continued, however, the data team were able to use routine healthcare data to complement data recorded by local staff by linkage to centrally held datasets generated as part of routine NHS care and national registries. (RECOVERY now harnesses over 25 different datasets from such routine data sources.) The extensive data linkage ensures very high completion of follow-up and avoids the risk of bedside data collection processes being overwhelmed during peaks. The use of routine healthcare datasets also allowed the ascertainment of outcomes which could not be recorded reliably by the local research team (for example when participants were transferred between hospitals to access intensive care facilities). These data systems also enabled the trial statistics team to report results soon after completing a treatment comparison. (For example, recruitment to dexamethasone was closed on 8 June with the preliminary results reported and widely publicised just 8 days later).

### Value of concise and timely statistical analysis reports to DMC

Collation and reconciliation of data were undertaken, blinded to treatment allocation, about 48 h before each scheduled DMC meeting. The data system included appropriate firewalls within the trial coordinating office to ensure there was no inadvertent unblinding of interim results to investigators. Two unblinded and non-voting statisticians (JRE and NS) are included on the DMC who each independently programmed and ran the analyses and then cross-checked results before each meeting (two unblinded statisticians were appointed partly to ensure that, should one of them fall ill with COVID, the other could continue). A DMC report should be a concise set of tables and graphs restricted to the information most relevant to the DMC’s decision-making process [15, 16]. For each intervention, our DMC report included just a handful of pages showing cumulative recruitment over time, baseline characteristics, treatments received during the admission period, 28-day Kaplan–Meier mortality curves (the primary outcome), and a detailed table presenting the effect of treatment allocation on all pre-specified efficacy and safety outcomes. As new interventions were added, so were additional relevant safety assessments. Subgroup analyses of the primary outcome (28-day mortality) by baseline characteristics were also provided as recruitment to each intervention matured, as were meta-analyses showing the interim RECOVERY results together with the results of all other available relevant trials.

### Confidentiality and importance of maintaining blinding of results in this open trial

The protocol states that *the Steering Committee, study staff, investigators, study participants, funders and other partners will remain blind to the interim results on study outcomes until 28 days after the last patient has been randomised for a particular intervention arm.* Maintaining blinding of interim DMC results is essential. Any deliberate or inadvertent release of unblinded interim results from the DMC could prejudice the successful completion of the trial and would certainly damage the perceived integrity of this crucial trial oversight body. However, there were several occasions when the UK regulatory agency, the MHRA, requested that the DMC should suspend recruitment or else provide unblinded safety information on the primary outcome of all-cause mortality for an ongoing comparison. We operated to the widely accepted principle that unblinded DMC analyses of the primary outcome and minutes would not be disclosed for regulatory or audit purposes, because *the regulatory review of data from a trial is most demonstrably objective when the regulatory scientists charged with the review have not been part of the unblinded monitoring process* [20]. If regulators (or other individuals outside the DMC) gained access to even part of the unblinded data, it might lead to inappropriate premature closure of particular treatment arms. Furthermore, the integrity of the trial could be undermined, especially if such information—inadvertently or otherwise—reached individuals with interests in the trial results, who could publish misleading inferences based on immature data. Hence, in the interests of patient safety, transparency, and scientific accountability, each request for unblinded data by the MHRA was handled promptly (often out of normal working hours) and resolved through discussion without revealing the unblinded interim primary results (see Additional file 1 for the details of these interactions). We did, however, on occasion permit the release of unblinded safety information on specific secondary safety outcomes to regulators. Confidential exchanges of *qualitative* information with other DMCs of trials testing similar treatments to those tested in RECOVERY were useful, however, when one or both committees were considering dropping a drug for safety concerns (see Additional file 1).

### Assessments of safety

In the RECOVERY trial, information collected relevant to safety included cause-specific and all-cause mortality (the primary outcome), use of invasive mechanical ventilation and renal replacement therapy, and specific adverse events relevant to the interventions being studied. For example, early in the first wave, when there were concerns about the potential for hydroxychloroquine to cause cardiac arrythmias, relevant fields were added to the 28-day outcome form. Similarly, when convalescent plasma and casirivimab + imdevimab were added as treatment arms, information about transfusion/infusion reactions was recorded, and when, later, aspirin was added, thrombotic and bleeding events were recorded. The RECOVERY eligibility criteria enabled the randomisation of pregnant women to several of the treatments being tested in the trial. Although relatively few pregnant women were included (98 in the first 2 years) and hence too few for any meaningful results in this one subpopulation alone, we were routinely presented with tabulations of all relevant outcomes among pregnant women and of their pregnancy outcomes. Children were also eligible for appropriate RECOVERY interventions, and during the first 2 years, 335 were randomised. We specifically reviewed outcomes for all children.

### ‘Competition’ for recruitment between arms, futility and the value of a factorial design

At the start of the pandemic, several treatments were prioritised for evaluation by the UK New and Emerging Respiratory Virus Threats Advisory Group. RECOVERY therefore utilised the advantages of a ‘multiarm’ design to allow the simultaneous evaluation of four treatments (lopinavir-ritonavir, dexamethasone, hydroxychloroquine, azithromycin) [21]. However, this also meant that these interventions were effectively ‘competing’ with each other for participants. The initial wave of the pandemic had a very sharp peak and hence daily recruitment declined rapidly after the first 8–10 weeks (with, at that point, no certainty about whether and to what extent there would be a resurgence in the UK). Hence, we needed to consider the trade-off between getting a slightly more convincing negative result for one high-profile treatment versus potentially recruiting more patients into another arm that could end up having a beneficial effect versus adding another treatment in its place while there were still large numbers of cases. For hydroxychloroquine and dexamethasone, given the uncertainties about effect sizes, the investigators had not initially specified any criteria for futility analyses. However, as the first wave of cases eased and recruitment slowed, the investigators (who at all times were blinded of any interim results) did ask us to review futility analyses for lopinavir-ritonavir and much later in the trial for colchicine (see Additional file 1 for details). In April 2020, when tocilizumab was added to the trial (and for most of the new interventions thereafter), interventions were added in a factorial manner, thus avoiding the problem of ‘competition’. This also had the beneficial effect for some of the later comparisons—corresponding to an expectation of more modest effect sizes—that much larger sample sizes could accrue and hence also provide information on combinations of treatments. For example, over Winter of 2020/2021, more than 10,000 patients were recruited into each of the factorial comparisons of aspirin and colchicine vs usual care without any impact on the ongoing recruitment of patients to convalescent plasma or casirivimab+imdevimab.

### Decision-making over the course of the trial

Our decision-making process was relatively straightforward for some of the interventions under study, but for others, specific issues arose that required considerable discussion to resolve. Four examples are summarised below. (Greater details on these decisions, and a summary of the more straightforward decisions, are detailed in Additional file 1).

#### Decisions regarding hydroxychloroquine

The early phase of the pandemic saw an intense public debate about the value of hydroxychloroquine in treating COVID-19 infection. On 28 March 2020, the US Food and Drug Administration (FDA) issued an Emergency Use Authorization (EUA) for treating COVID-19 patients with hydroxychloroquine, but reliable randomised evidence was urgently needed. By 13 May 2020, when 1387 patients had been allocated to hydroxychloroquine and 2813 to usual care, the effect of allocation to hydroxychloroquine showed a small non-significant increase in 28-day mortality and fewer patients discharged alive from the hospital within 28 days. Given our terms of reference (particularly the need to provide evidence that was strong enough to affect national and global treatment strategies) and the trial steering committees planned sample size of at least 2000 in the hydroxychloroquine arm, we saw no cogent reason to modify the protocol. However, we had some concerns about this pattern; concerns of the type described by DeMets as an ‘agonising negative trend’, where the best option is often to allow more data to accumulate [22]. Shortly after that meeting, we came under pressure from the MHRA to terminate recruitment before a reliable estimate of the treatment effect was possible. A report in the Lancet on 22 May of a large non-randomised multinational registry of patients admitted to hospitals with COVID-19 had estimated that the use of hydroxychloroquine was associated with 34% increased mortality (HR 1.34, 95% CI 1.22 to 1.46) after adjustment for multiple measured confounders [23]. The MHRA wrote to the principal investigators, stating that recruitment to the hydroxychloroquine arm stop immediately (the Lancet report was later retracted, but not until 5 June 2020 [23]). The principal investigators requested that we urgently review the current data. After much discussion and interaction with MHRA, over a public holiday weekend, during which the MHRA requested but were not provided with, unblinded analyses of the primary outcome, recruitment was allowed to continue. On 4 June, the observed effects on mortality were such that even modest benefit was effectively excluded, even without a formal analysis of futility, and so we advised the investigators should be unblinded to the results for hydroxychloroquine (additional details of the events between 22 May and the announcement on 5 June are described in the Additional file 1). The principal investigators closed recruitment to the hydroxychloroquine arm and announced the preliminary results the next day [24]. The FDA revoked its EUA for hydroxychloroquine on June 15, 2020, and national and international guidelines were promptly updated thereafter.

#### Decisions regarding dexamethasone

As the number of patients allocated dexamethasone increased towards its planned sample size of 2000 patients, the trend to an overall reduction in 28-day mortality and an increase in the proportion discharged alive from the hospital began to emerge. By the DMC meeting on 28 May, when data was available for 1998 patients allocated dexamethasone, although allocation to dexamethasone was associated with a 14% reduction in 28-day mortality 2p = 0.0098, there was an apparent qualitative interaction by baseline oxygen requirement, suggesting significant harm in patients not requiring supplemental oxygen at baseline. It was therefore important that follow-up should be complete before unblinding the trial team and making any public announcement. The investigators closed recruitment to the dexamethasone arm on 8 June 2020 when it reached its planned sample size of 2000. Analysis of the final data revealed that dexamethasone significantly reduced 28-day mortality by about one-fifth for patients requiring supplemental oxygen at randomisation and by about one-third for patients who required invasive mechanical ventilation at randomisation, but did not reduce mortality among patients who required no supplemental oxygen at randomisation [4]. The findings were announced on 16 June 2020 [25, 26] and had an immediate worldwide impact on the treatment of the sickest patients with COVID-19.

#### *Decisions regarding a high-risk subgroup randomised to casirivimab* + *imdevimab*

On Friday 30 October 2020, Regeneron Pharmaceuticals notified the RECOVERY investigators that the independent Data Safety Monitoring Board of their COV-2066 trial testing the monoclonal 'antibody cocktail' casirivimab+imdevimab had advised pausing recruitment, for any hospitalised patients requiring high-flow oxygen or ventilation, because of a potential safety issue. The investigators informed the MHRA and immediately issued a statement indicating that RECOVERY would continue to enrol as planned but that the full DMC would review the data 6 days later on 5 November 2020, at which we recommended recruitment to continue. A series of exchanges then followed among the MHRA, the investigators and us as to whether we should be obliged to release the unblinded primary efficacy and safety data to the MHRA (see Additional file 1). A confidential discussion between the RECOVERY DMC Chair and DMC chair of the COV-2066 trial on 7 November enabled the RECOVERY DMC to recommend the continuation of recruitment to all subgroups in the casirivimab + imdevimab arm. Despite this, the MHRA continued to request access to the unblinded data which supported that decision. After further correspondence, clarifying the list of outcomes reviewed and the number of patients and deaths overall and in the subgroup of concern (for both treatment groups combined), the MHRA accepted our position not to supply unblinded primary outcome data. The exchange of correspondence that took place during this period highlights how important it is to avoid outside influence on DMC decisions, yet it is also possible to reassure regulatory bodies charged with protecting the public’s interest, while maintaining confidentiality, protecting the integrity of the data and preserving the DMC’s decision-making process. When the final results were available, these showed consistent benefit among seronegative patients, regardless of the level of respiratory support received at baseline [12].

#### Decisions regarding how to act following a press release about tocilizumab

On 19 November 2020, the day of the scheduled RECOVERY DMC meeting, the REMAP-CAP investigators issued a press release stating that, among the first 303 patients randomised to receive immune modulation treatments (tocilizumab, sarilumab, anakinra, interferon, or no immune modulator), tocilizumab was associated with better outcomes compared to no immune modulation with a high degree of statistical certainty (99.75% probability that tocilizumab was superior to no immune modulation); the nature and size of the apparent benefit were not stated. At that point, the tocilizumab arm of RECOVERY had recruited 1858 patients (with 438 deaths); in light of these data and given the lack of detail in the press release, we recommended continuing recruitment. For the DMC meeting on 17 December 2020, the RECOVERY investigators informed us that they were not only seeking a clear answer overall but also in the subgroup of patients receiving dexamethasone. At this meeting, our report also included a presentation of the RECOVERY result in the context of a meta-analysis of all the available randomised trials of tocilizumab. As the totality of the evidence was inconclusive it was clear that RECOVERY needed to reach its planned sample size to provide a clear answer, we again recommended continuing recruitment. The final results based on 4116 patients reliably confirmed the mortality results from REMAP-CAP, but importantly also confirmed the benefit among patients receiving dexamethasone [9].

### Summary

The burden of responsibilities carried by a DMC is well-known and is even greater in the context of a pandemic [3, 27]. The responsibility rested with the RECOVERY trial DMC to balance caution against speed: caution to wait for sufficient evidence to accumulate for robust conclusions and speed to determine which treatments were clearly ineffective and which clearly beneficial. The need was to inform the care of millions of COVID-19 patients worldwide at the earliest opportunity. The responsibility for such a high-profile trial in such a dominant news area brought additional pressures, for example, the need to react swiftly to interim results of other (usually much smaller and less informative) trials, often in the limited form of a press release or of non-randomised studies published in high-profile journals. The intensity of the work required to achieve timely well-informed decision-making by the DMC placed a considerable burden on all members of the trial team as well as the DMC members. The need for the DMC to ‘keep its nerve’ when under pressure has been mentioned in the context of trials in neonatal medicine [28]; and this was certainly the case for us. We had, on several occasions, to respond urgently to demands from regulatory authorities to release unblinded results; on each occasion, we were, after considerable effort, able to provide information to address their concerns, without the need to provide unblinded results and hence we were able to preserve the integrity and confidentiality of the data.

## Supplementary Information

**Additional file 1. **Online Appendix.

## Data Availability

The trial protocol, DMC charter, and other materials are available at https://www.recoverytrial.net/
