# Supplementary material for: Experiences of the Data Monitoring Committee for the RECOVERY trial, a large-scale adaptive platform randomised trial of treatments for patients hospitalised with COVID-19
Source: Trials. 2022 Oct 18;23:881. doi: 10.1186/s13063-022-06824-6 (PMC9579551; doi:10.1186/s13063-022-06824-6)
Supplement: Supplementary file 1 — Additional file 1. Online Appendix. [file 13063_2022_6824_MOESM1_ESM.pdf]

Experiences of the Data Monitoring Committee for the RECOVERY trial, a large-scale adaptive platform randomised trial of treatments for patients hospitalised with COVID-19

Online Appendix

Peter AG Sandercock, Janet Darbyshire, David DeMets, Robert Fowler, David G Lalloo, Mohammed Munavvar, Natalie Staplin, Adilia Warris, Janet Wittes, Jonathan R Emberson

Contents Page

Timeline of key events over the first 2 years of RECOVERY 2

Version 1.1 of the Data Monitoring Committee Charter 9

Timeline of key events over the first 2 years of RECOVERY

9th March 2020. Chief investigators begin drafting first version of the protocol

13th March 2020. Protocol submitted for MHRA and ethics approval
The protocol included details of the DMC guidance and the names of all the members.

19th March 2020. First patient randomised.

28th March 2020. The US FDA issue an Emergency Use Authorization (EUA) for treating COVID-19 patients with hydroxychloroquine

22nd May 2020. Non-randomised study of COVID patients published in the Lancet suggests that hydroxychloroquine may increase mortality by one third

23rd May 2020: Urgent DMC review of hydroxychloroquine
In response to an MHRA demand to close recruitment, the principal investigators asked the DMC to undertake an urgent review of the data. An interim analysis on the morning of Saturday 23rd May 2020 was performed, based on 1515 patients allocated hydroxychloroquine plus standard care versus 3060 allocated standard care alone. After reviewing these data, the DMC unanimously agreed that there was no cogent reason to suspend recruitment to hydroxychloroquine because of safety concerns. In particular, the DMC noted that the current observed hazard ratio (HR) for 28-day mortality (for hydroxychloroquine vs usual care) was significantly different (at 2p=0.01) to the HR of 1.34 from the paper by Mehra *et al*. The MHRA accepted this and agreed recruitment could continue.

28th May 2020: The following DMC meeting
The chief investigator's report to the DMC included the following guidance:

'Since the last full meeting, we considered our plans for the completion of enrolment to the various main arms of the trial. Based on the current [blinded] rate for the primary outcome of 28-day mortality (24.7%), a comparison of 2,000 patients on active treatment vs. 4,000 patients allocated standard of care alone, would have 90% power at 2p=0.01 to detect a proportional reduction of 18% (e.g. 25% vs. 20.5%). Our current intention is to continue each of the main treatment arms until at least 2,000 patients have been allocated to the active treatment. However, we are not seeking any specific guidance on stopping for futility from the Data Monitoring Committee at this meeting.

By 28th May 2020, the RECOVERY team had identified more than 30 ongoing apparently randomised trials of chloroquine or hydroxychloroquine and details were made available to the DMC. Notably, the WHO SOLIDARITY trial (which had by then randomised about 3500 patients) had suspended recruitment to hydroxychloroquine on 24th May 2020. The French trial (DISCOVERY) had stopped a few weeks earlier due to lack of enrolment; data from that trial were to be included in SOLIDARITY. A Cochrane review (circulated to the DMC) commissioned by the WHO concluded that the current published evidence regarding the effects of chloroquine or hydroxychloroquine was of low or very low certainty. After review of the unblinded data, the DMC again agreed that there was no cogent reason to suspend recruitment to hydroxychloroquine because of safety concerns. The DMC formal report to that effect was forwarded to MHRA and made publicly available.

The DMC also discussed the request from the MHRA for unblinded analyses of the primary outcome. The Committee was sympathetic to the concerns expressed by MHRA about the safety of hydroxychloroquine, but unanimously agreed that it was not appropriate for either the investigators or the independent DMC to release unblinded data at a time when: a) the hydroxychloroquine arm had yet to reach an appropriately powered sample size; and b) the DMC felt the safety and efficacy data did not meet pre-stated criteria for unblinding and informing the trial Steering Committee.

29th May 2020: Wider concerns expressed by the MHRA about the RECOVERY trial

On the 29th May 2020, the Chair of the DMC was contacted by the Chair of the UK Commission for Human Medicines (CHM) (the CHM provides expert advice to the MHRA on matters of drug safety and efficacy). During this informal conversation, it was immediately clear that CHM Chair wanted to help resolve matters between the DMC and the regulators in a straightforward and constructive way. Apparently, the MHRA and CHM had not seen a copy of the DMC charter and were unclear about the nature and frequency of meetings. The CHM sought clarification on a few points: the level of experience of the DMC; the expectation that, in this adaptive trial with 10,000 patients already randomised, at least one 'unpromising' arm would have been dropped; and details of the DMC stopping rules (Note: Details of the DMC Membership, meeting frequency, and stopping guidance was included in every version of the protocol submitted to and approved by MHRA since the start). The DMC Chair clarified that the DMC members collectively had enormous experience in all relevant domains and set out what happened in the lead up to the decision to continue all arms. In particular, it was clarified that the chief investigators had confirmed their wish to recruit at least 2000 patients to each active arm (unless 'the randomised comparisons in the study have provided evidence on mortality that is strong enough (with a range of uncertainty around the results that is narrow enough) to affect national and global treatment strategies' as specified in the protocol and DMC charter) and that they did not want the DMC to consider stopping for futility (in order to provide as reliable a result as possible for this drug).

1st June 2020: Agreeing a constructive response to the MHRA request

The DMC Chair offered to speak to the MHRA on behalf of the RECOVERY trial DMC, on condition that the meeting would be in strict confidence and would not involve the release of unblinded data. In preparation for the meeting, the chief investigators submitted an extensive dossier to MHRA/CHM including: the external evidence the DMC had reviewed; the DMC Charter, key items of correspondence that set out the points above, and blinded data on event rates. The DMC Chair joined a meeting of the CHM on 1st June 2020 and was able to address the committee's concerns.

4th June 2020: Further urgent request for unblinded data from MHRA

Although the MHRA had agreed that RECOVERY was not required to suspend randomisation to hydroxychloroquine, at 12.24 on 4th June they wrote to the chief investigators stating that:

'It is seen as critical to have the following information from RECOVERY by close of business today … of the current hazard ratio for all-cause mortality at 28 days and the associated 95% confidence interval … from the HCQ and the standard care arms supported by data on the number and proportion of individuals in each group who had died at 28 days'.

Furthermore, if the Sponsor (i.e., the University of Oxford) did not comply with the MHRA's requests, then the MHRA could force closure of the hydroxychloroquine arm if they wished (or, indeed, could force closure of the whole trial). The chief investigators and the DMC agreed that release of unblinded data to the regulator was not appropriate. To formulate an appropriate response, the DMC chair and DMC statistician reviewed both an updated set of analyses on the 11,048 patients recruited by 4th June 2020 and the relevant external evidence. By then, the Lancet had retracted the Mehra paper, which had initially triggered the MHRA's concerns. It was decided the full DMC should review the updated analyses immediately. As the MHRA had set a deadline to receive the data by the end of the day, the updated analyses were circulated to all members of the DMC. The DMC Chair then contacted each member by telephone to seek their opinion on the most appropriate course of action, asking for written comments. In summary, the DMC noted that the dexamethasone arm had reached its target sample size and the chief investigators had already informed the DMC that they planned to close recruitment. The hydroxychloroquine comparison included 1542 allocated hydroxychloroquine and 3132 controls. The observed effects on mortality were now such that even modest benefit was effectively excluded. The DMC was unanimous that any release of data should be to the chief investigators and not to the regulator. The DMC considered it likely that, when they saw these interim results, the chief investigators would decide to close recruitment to the hydroxychloroquine arm (as

well as their planned closure of the dexamethasone arm), with a view to completing data for those two arms and reporting the results as soon as practicable. The DMC agreed that all other treatment arms continue without interruption. The DMC Chair circulated a draft report to all DMC members by email. When all DMC members had approved the report, the DMC recommendation letter was forwarded to the chief investigators. Recruitment to the hydroxychloroquine closed the following day (Friday 5th June) and the dexamethasone arm closed on Monday 8th June. The DMC recommended that the chief investigators should look at **both** the hydroxychloroquine and dexamethasone results (without waiting for complete follow-up on all participants).

5th June 2020: Preliminary hydroxychloroquine results announced

16th June 2020: Dexamethasone results announced
The chief investigators undertook further analyses of the dexamethasone data, to ensure the evidence of benefit was robust and to fully understand any variations in effect across key subgroups, before making an announcement.

29th June 2020: Lopinavir-ritonavir result announced
By the DMC meeting on the 25th June 2020, the WHO had stopped recruitment into the SOLIDARITY lopinavir arm and announced that interim results would be available the following week. 4972 patients had been recruited to the lopinavir-ritonavir arm in RECOVERY. The RECOVERY investigators informed the DMC that, in the light of the low recruitment rate it was likely to take 1-2 more months before reaching the original target of 2000 vs 4000, and requested that an assessment of futility be done. The DMC analyses showed that the conditional power to demonstrate a significant reduction in mortality was negligible. The DMC recommended that the chief investigators should be unblinded to the current lopinavir results in the expectation that they would cease recruitment on the grounds of futility (which they did then do).

30th October 2020. **Announcement by COV-2066 raises concerns about the casirimivab+imdevimab arm**
The chief investigators wrote to all study sites:

'Today Regeneron have issued a statement announcing a hold on recruitment of COVID-19 patients with high oxygen requirements to their trial of the REGN-CoV-2 antibody cocktail. We note that this is a hold pending collection and analysis of further data, and that enrolment and randomisation of other patients with COVID-19 to studies of this drug continues. Since REGN-CoV-2 is being studied in the RECOVERY trial, we have today discussed this announcement with Regeneron, with the UK Medicines and Healthcare products Regulatory Authority, and with the RECOVERY independent Data Monitoring Committee. The independent Data Monitoring Committee will review the latest data from the ongoing RECOVERY trial on Thursday 5th November. In the meantime, the RECOVERY trial will continue as planned.'

5th November 2020. The DMC reviewed the data for casirimivab+imdevimab (total n=325, 14 deaths) and there were no safety concerns based on the limited data available (including in the subset on oxygen with ventilatory support or requiring invasive mechanical ventilation). The committee saw no reason to suspend recruitment in RECOVERY to this arm and issued a letter to that effect. The next meeting was scheduled for 19th November 2020.

6th November 2020. The MHRA wrote to the chief investigators:

'*MHRA currently does not have sufficient information to be able to be satisfied about patient safety for those on high flow oxygen or being mechanically ventilated in the Regeneron arm of RECOVERY. For your information we are in contact with both FDA and Regeneron and will still require a robust and detailed justification for not halting these patients in RECOVERY. The outcome letter from the DMC meeting yesterday is not sufficient without some supporting information, based on what we are aware of for the US trial.'*

7th November 2020. The Chair of the RECOVERY DMC made contact with, and then met by teleconference, the Chair of the Regeneron trials DSMB, which oversaw all the casirimivab+imdevimab trials in both outpatients and inpatients. He confirmed there were no safety concerns in the outpatient trials. About 750 patients had been recruited in the inpatient study COV-2066; 250 in each of the three arms (placebo, lower dose casirimivab+imdevimab, higher dose casirimivab+imdevimab). The safety concern arose in the subset of about 210 patients (i.e., 70 in each arm) requiring either high-intensity oxygen or mechanical ventilation at the time of their randomisation. At the time the Regeneron trial's DSMB met to review the data in this subgroup, there were about 48 deaths split among the three arms with an apparent adverse dose-relationship. At this stage, the efficacy data were scant as many patients had not reached the time points for the measurement of efficacy. After the date of that analysis, however, further deaths had accrued in the placebo arm, so that when the Regeneron Trials DSMB Chair received an updated analysis, the dose-effect on death (the chief cause of the safety concern) was diminished (now about 55 deaths across all 3 groups); the efficacy data was still too immature to make estimates of efficacy reliably. The two DMC Chairs discussed what had been observed in the RECOVERY casirimivab+imdevimab arm overall and in the subset requiring either high-intensity oxygen or mechanical ventilation. The Regeneron Trials DSMB Chair was supportive of the RECOVERY DMC's decision to continue recruitment.

The MHRA accepted the decision to continue recruitment, but the chief investigators were asked to provide the conclusion of the DMC meeting to be held on November 19th, clarification that the DMC specifically considered the data for the Regeneron antibody specifically in patients on high flow oxygen or on mechanical ventilation and data for the Regeneron antibody in all patients, including deaths in the relevant groups, as supporting evidence to justify the DMC decision on November 19th. These items were duly sent to, and accepted by, the MHRA.

19th November 2020. Scheduled DMC meeting and announcement by REMAP-CAP that tocilizumab is superior to no immune modulation

On the morning of 19th November 2020, prior to the DMC meeting later that day, the REMAP-CAP investigators announced the superiority of tocilizumab compared with no immune modulation. The tocilizumab safety and efficacy data from RECOVERY were reviewed later that day, together with a meta-analysis of all the randomised trials with available mortality data. As the totality of the evidence was inconclusive, it was clear that RECOVERY needed to reach its planned sample size of 4000 to provide a clear answer. At the same meeting, the DMC reviewed the data for the 766 patients in the casirimivab+imdevimab comparison. In the light of these data, all available external information and the considerations specified by the MHRA, the DMC saw no cogent reason to modify the protocol or intake to the study and recommended continuing recruitment of eligible patients to all active arms of the trial.

20th November 2020. The MHRA again requested unblinded primary outcome data on casirimivab+imdevimab. The chief investigator replied to that request as follows:

'I really think we must all trust in the expertise of the independent Data Monitoring Committee. Indeed the membership of this DMC is extremely strong with decades of experience in clinical trials, relevant aspects of medicine, statistics, and data monitoring committee activity. Several of these members have played key roles with (or even within) FDA and MHRA in the past (a list of the DMC members names and areas of expertise was included here).

They were aware of the situation in the Regeneron trial. They then reviewed the unblinded data on the larger group of patients in RECOVERY and reached a recommendation. Furthermore, they have said that they will hold a further review in 13 days' time. It is also worth noting that this is not a treatment that is available as part of routine NHS care.

The reason why clinical outcome trials such as RECOVERY have an independent Data Monitoring Committee is to provide ongoing assessments of the safety of the treatment whilst

protecting the integrity of the results (which will inform the care of future patients) by avoiding the need to disclose unblinded information on study outcomes (except in the case that there is actionable evidence of benefit or hazard). This approach is enshrined in guidelines issued by ICH and many others.'

24th November 2020: The MHRA replied:

'Thank you for the clarification. Our current expectation for RECOVERY is that the MHRA should continue to be informed of the DMC decisions, including the following explicit confirmation for the Regeneron antibody arm: that other external data (if available) has been considered in any decision; mortality data for the active and control arm have explicitly been reviewed; the data has also been reviewed in terms of any differences in safety or outcome between not just active and control but also between those on low flow/no oxygen and those on high flow oxygen/ventilated.'

This information was provided to MHRA for each subsequent DMC meeting and accepted, avoiding the need for the release of any unblinded results.

14th December 2020: Azithromycin result announced

As the data on azithromycin accumulated, no concerns arose about its safety, but equally no hint of benefit emerged. The DMC did not recommend change in recruitment plans. The chief investigators closed recruitment to the azithromycin arm on 27th November 2020, when 7763 patients had been recruited (2582 allocated azithromycin and 5181 allocated usual care) and near-complete 28-day mortality data were available. The preliminary results were announced on 14th December. Although azithromycin never seemed a particularly promising (or harmful) treatment, for most of the period it was active in the trial it was not in direct competition against any other treatment (as lopinavir-ritonavir, dexamethasone and hydroxychloroquine had all completed by mid-2020 while other treatments assessed in the second half of 2020 were all included as factorial assessments). This lack of 'competition' with other interventions made it fairly straightforward for the DMC to allow the azithromycin comparison to continue to the final sample size determined by the Steering Committee.

7th January 2021: Recommendation to pause recruitment to convalescent plasma for patients on invasive mechanical ventilation or ECMO

Convalescent plasma, like hydroxychloroquine the previous year, had very strong proponents encouraging its use. The chief investigators' initial aim was to identify an overall treatment effect irrespective of baseline antibody status. However, during 2020 evidence emerged that baseline antibody titre was an important predictor of prognosis and, more importantly, potentially also a predictor of response. Given the possibility of a beneficial effect being limited to those who were seronegative at randomisation, the trial Steering Committee determined that a large sample size was going to be needed (not least because, if convalescent plasma were useful even in a subset of patients, then its low cost and wide availability could have worldwide implications for the treatment of patients with COVID-19).

Despite no reliable randomised evidence at the time, over 100,000 US patients with COVID 19 were given convalescent plasma under the US FDA's Expanded Access Program. In preparation for the DMC meeting on 7th January 2021, the RECOVERY investigators wrote to the DMC**:**

'As discussed previously, it is reasonable to anticipate (and many experts in this field believe) that the effect of convalescent plasma will be larger among those patients who have not (yet) mounted a good antibody response for themselves …We are seeking a compelling answer that will be widely accepted and adopted internationally.'

The DMC noted that a similar differential subgroup response by baseline antibody status was anticipated for the Regeneron monoclonal antibody cocktail: see below.

During the DMC Meeting on 7th January 2021, data for 8607 patients (including 1493 deaths) were reviewed. Although there was no overall evidence of benefit for any outcome, it remained plausible that some benefit could exist for those with low antibody titre. However, a trend towards increased mortality was seen in the subset of 524 patients (6%) on invasive mechanical ventilation or ECMO at randomisation. This was concerning given an announcement by the REMAP-CAP investigators earlier in the day that they had stopped recruitment to their convalescent plasma comparison for patients admitted to ICU who required organ support with one or more of non-invasive or invasive ventilator support, or receipt of infusion of vasopressor or inotropes. The RECOVERY DMC Chair spoke by teleconference with the Chair and statistician of the REMAP-CAP DMC. During this discussion, it was clarified that the decision to stop recruitment in REMAP-CAP was made for futility (rather than because of any safety concern).

The RECOVERY DMC subsequently reconvened (at 7pm UK time on January 7th), reviewed the *combined* evidence on mortality from RECOVERY and REMAP-CAP, and agreed that recruitment to the convalescent plasma comparison of patients on invasive mechanical ventilation or ECMO should be paused in RECOVERY (but that recruitment of all other patient groups should continue). This was implemented the next day.

14th January 2021: Further deliberations on convalescent plasma

The RECOVERY DMC met again on 14th January 2021 to review the data for convalescent plasma (now with 10,406 patients and 1873 deaths). The overall effect on 28-day mortality was now non-significantly *adverse,* with a suggestion of *increased* mortality among those with baseline antibody titre ≥8M units with little to no benefit among those with baseline antibody titre <8M units. Given that, due to the very rapid recent recruitment to this comparison, complete follow-up of all currently randomised patients would provide a good test of the hypothesis of that the effects of convalescent plasma differed materially in any given subgroup, the DMC recommended that recruitment to the convalescent plasma comparison should cease. The chief investigators accordingly closed recruitment to convalescent plasma.

15th January 2021: Convalescent plasma results announced

11th February 2021: Tocilizumab result announced

The Steering Committee closed the tocilizumab arm on 25th January 2021 when it reached its planned sample size and announced the results on 11th February**.**

4th March 2021: DMC meeting – Colchicine and aspirin

The chief investigators informed the DMC that for both the colchicine and aspirin comparisons they wanted to have 90% power at 2p=0.01 to detect a 12.5% proportional reduction in 28-day mortality. This would require around 15,000 patients for each comparison. At the DMC meeting on 4th March 2021, 11,162 patients had been recruited to the colchicine comparison and 14,167 to the aspirin comparison. For colchicine, the DMC took the view that it was now sufficiently clear that colchicine was ineffective and that the results were already strong enough to influence global policy (and a formal futility analysis reinforced that it would not be worthwhile continuing to recruit patients to the colchicine arm). The DMC therefore recommended ceasing recruitment to the colchicine arm.

5th March 2021: Colchicine results announced

21st March 2021: Principal investigators close recruitment to aspirin comparison

Recruitment closed as the planned sample size had been achieved**.**

27th April 2021 Steering Committee decides to close recruitment to casirimivab+imdevimab.

By that date, over 9700 patients had been randomised and recruitment was very slow. At its meeting that day, the Trial Steering Committee decided that recruitment should close. This decision was enacted the following week after the statistical analysis plan for this comparison (which was to be

used to support any regulatory filing) had been modified to place adequate emphasis on the group of patients who were seronegative at the point of randomisation.

8th June 2021: Aspirin results announced
The results were announced as soon as 28 day follow-up was available, and published on a pre-print server.

16th June 2021: Casirivimab+imdemimab results announced

29th December 2021: Baricitinib recruitment closed
Recruitment closed as the planned sample size had been achieved**.**

3rd March 2022: Baricitinib results announced

Randomised Evaluation of COVID-19 Therapy (RECOVERY)

Data Monitoring Committee Charter

Version 1.1

Table of Contents

1 STUDY DETAILS **2**

2 BACKGROUND **2**

3 ROLES AND RESPONSIBILITIES **2**

4 APPOINTMENT AND MEMBERSHIP **3**

4.1 DMC MEMBERS 3
4.2 AD HOC DMC ADVISORS 3
4.3 MODIFICATIONS TO DMC MEMBERSHIP 3
4.4 CONFLICTS OF INTEREST 3

5 REVIEW OF EMERGING INFORMATION **3**

5.1 FREQUENCY OF REVIEWS 3
5.2 GUIDELINES FOR RECOMMENDING CHANGES TO THE PROTOCOL 4
5.3 AD HOC REVIEW OF SUSPECTED SERIOUS ADVERSE REACTIONS 4
5.4 REVIEW OF INFORMATION FROM EXTERNAL SOURCES 4
5.5 DECISION MAKING 4

6 PUBLICATIONS **4**

7 INDEMNITY **5**

8 CONDUCT OF MEETINGS **5**

8.1 AGENDA 5
8.2 MINUTES AND ARCHIVING 5
8.3 COMMUNICATION OF DMC MEETING OUTCOME 5

9 APPENDIX: KEY CONTACT DETAILS [CONFIDENTIAL] **6**

9.1 DATA MONITORING COMMITTEE 6
9.2 STEERING COMMITTEE 6

Version History

Version	Date	Summary
1.0	15-Apr-2020	Approved by DMC
1.1	21-Dec-2021	Updated membership

(i) Study Details

Title:	Randomised Evaluation of COVID-19 Therapy (RECOVERY)
EUDRACT number:	2020-001113-21
Sponsor:	University of Oxford
Funder:	UK Research & Innovation / National Institute for Health Research
Principal Investigators	Prof Peter Horby & Prof Martin Landray (University of Oxford)

(ii)Background

RECOVERY is a randomised controlled trial among hospitalised adults hospitalised investigating the effects of different therapies for COVID-19 disease. To facilitate collaboration, even in hospitals that suddenly become overloaded, patient enrolment, follow-up, and all other trial procedures are greatly streamlined.

It is anticipated that many thousands of individuals will be randomly allocated between several treatment arms, each to be given in addition to the usual standard of care in the participating hospital. Initially, these treatment arms will be: No additional treatment *vs* Lopinavir-Ritonavir *vs* low-dose corticosteroids (dexamethasone) *vs* Hydroxychloroquine. (An additional arm of nebulised interferon has provisional approval but is not active at present.) For patients for whom not all the trial arms are appropriate or at locations where not all are available, randomisation will be between fewer arms. New trial arms may be added as evidence emerges that other candidate therapeutics should be evaluated. The main outcomes are in-hospital death, discharge, use of ventilation and use of renal dialysis or haemofiltration.

This document describes the roles and responsibilities of the independent Data Monitoring Committee (DMC) and provides guidelines for their decision-making.

(iii) Roles and Responsibilities

The independent DMC will act in an advisory capacity to the Steering Committee. Responsibilities of the DMC are:

(i) to provide an independent overview of the safety of trial participants; and
(ii) to make recommendations about continuation, termination or other modifications to the trial protocol (in particular for individual arms) based on their unblinded review of the study clinical outcome data.

The DMC will function independently of all other individuals and bodies associated with the RECOVERY trial, including the Investigators, the Steering Committee, and the Funder.

(iv) Appointment and Membership

a. DMC Members

On the invitation of the Steering Committee Chair and the Principal Investigators, the following have agreed to serve as members of the DMC:

Name	Role	Location	Expertise
Peter Sandercock	Chair	Edinburgh, UK	Clinical trials in stroke medicine
Janet Darbyshire	Member	London, UK	Clinical trials in infectious disease
Robert Fowler	Member	Toronto, Canada	Clinical trials in critical care
David Lalloo	Member	Liverpool, UK	Clinical trials in infectious disease
David DeMets	Member	Wisconsin, USA	Clinical trial statistician
Mohammed Munavvar	Member	Preston, UK	Respiratory medicine
Adilia Warris	Member	Exeter, UK	Paediatrics
Janet Wittes	Member	Washington DC, USA	Clinical trial statistician

Prof Jonathan Emberson and Dr Natalie Staplin (Nuffield Department of Population Health, University of Oxford) are the statisticians to the DMC. They will be responsible for providing the unblinded report to the DMC and for drafting the minutes of its meetings.

b. Ad Hoc DMC Advisors

If required by the DMC Chair, after discussion with other members of the DMC, experts in a particular field (e.g. ventilation, infectious disease) may be asked to provide additional advice in confidence on specific aspects of the unblinded data. Not only would the content of such discussions with DMC Advisors be confidential, but every effort should be made to ensure that the fact that their advice has been sought also remains confidential.

c. Modifications to DMC Membership

Further DMC Members may be appointed if members resign or are unable to attend for a prolonged period (e.g. because of illness), or at the request of the DMC Chair. All such appointments must be agreed by the Principal Investigators.

d. Conflicts of Interest

The DMC Members and Advisors must not otherwise be involved in the conduct of the RECOVERY trial and must have no significant financial or other conflicts of interest with the Sponsor. In particular, no DMC Member or Advisor should have a financial investment in the manufacturers of any of the study treatments that would result in questions regarding his/her independence.

All DMC Members and Advisors should disclose any conflict of interest to the DMC Chair at the start of each meeting thereafter.

(v)Review of emerging information

a. Frequency of reviews

During the study, interim analyses of all study data will be supplied in strict confidence to the independent DMC. The DMC will request such analyses at a frequency relevant to the emerging data from this and other studies (typically every 2-4 weeks). Additional meetings of the DMC may be called at any time by the Chair or at the request of the Principal Investigators.

b. Guidelines for recommending changes to the protocol

At each review, the DMC will independently evaluate the study data and any other information considered relevant.

The DMC will determine if, in their view, the randomised comparisons in the study have provided evidence on mortality that is strong enough (with a range of uncertainty around the results that is narrow enough) to affect national and global treatment strategies.

In such a circumstance, the DMC will inform the Trial Steering Committee who will make the results available to the public and amend the trial arms accordingly.

Unless this happens, the Steering Committee, study staff, investigators, study participants, funders and other partners will remain blind to the interim results on study outcomes until 28 days after the last patient has been randomised for a particular intervention arm (at which point analyses may be conducted comparing that arm with the no additional treatment arm).

c. Ad Hoc Review of Suspected Serious Adverse Reactions

Individual unblinded reports of each Suspected Serious Adverse Reaction (SSAR) will be provided to the DMC Chair by Central Coordinating Office clinicians. These may be provided at the time of the next meeting, or sooner if deemed clinically necessary.

d. Review of Information from External Sources

During the RECOVERY trial, there may be new information pertaining to the safety and efficacy of the study treatments, including results from other trials of similar or related treatments. The Principal Investigators will review any such information and make a written assessment for consideration by the DMC of whether, in its view: (i) any changes should be made to the trial protocol; and/or (ii) further information should be provided to investigators.

It may be desirable during the RECOVERY trial to combine or compare its results with those of other similar ongoing trials. If the DMC Chair wishes the interim data from RECOVERY to be shared with another trial they will first discuss this with the Steering Committee chair and Principal Investigators. Any sharing of data will be done in a way which minimises any risk to the integrity of the RECOVERY trial.

e. Decision making

In general, DMC decisions should be made by consensus, but where this is not possible, decisions will require a simple majority of the voting members of the DMC present at the meeting. For the avoidance of doubt, the DMC Statisticians and any ad hoc advisors are non-voting.

(vi) Publications

The Steering Committee will be responsible for drafting the main reports from the study and for review of any other reports. DMC Members will be given the opportunity to provide comments on the main results manuscript prior to its submission for publication.

(vii) Indemnity

The University of Oxford will indemnify the DMC members against any claims or legal actions for performing the role outlined in the DMC Charter. The details of the indemnity will be outlined in detail in the agreements between the University and the DMC Member (or their institution).

(viii) Conduct of meetings

a. Agenda

Session	Present	Content
**Open***	DMC members<br>DMC statistician<br>Principal Investigators<br>Study statisticians	Principal Investigators to report on:<br>• Data quality, and completeness of follow-up and adjudication<br>• Any relevant new external evidence (especially results from other relevant ongoing trials)<br>• Any proposals for changes in the study protocol
**Closed**	DMC members<br>DMC statisticians	• DMC Chair to review conflict of interest statements from all DMC members<br>• DMC statistician to report on unblinded data<br>• DMC to formulate recommendations
**Open***	DMC members<br>DMC statistician<br>Principal Investigators<br>Study statisticians	• Principal Investigators to clarify any issues raised<br>• Discussion of any action items<br>• Set date of next meeting

* No unblinded information is to be presented or discussed during the Open sessions.

b. Minutes and Archiving

Unless otherwise determined by the DMC Chair, minutes for the Open and Closed sessions will be taken by the DMC Statistician. The DMC Chair will be responsible for the accuracy of the minutes, and for their secure storage until the end of the trial.

Meeting documents (including closed session minutes) will only be distributed by secure means (e.g. encrypted and password-protected electronic files). These will remain confidential until the end of the trial, at which time they will be archived by the Sponsor and may be made available for public scrutiny (e.g. by regulatory authorities). Any hard copy meeting documents or closed session minutes must be disposed of securely after each meeting.

c. Communication of DMC Meeting outcome

Within 1 week after each meeting, the DMC Chair will provide the Chief Investigator with a letter stating the general outcome of the meeting and any recommendations. For example, this letter may simply contain the statement that the trial should continue as planned and give the planned date for the next DMC meeting. Unless indicated otherwise by the DMC Chair, this letter will not be considered confidential.

(ix) APPENDIX: Key contact details [CONFIDENTIAL]

a. Data Monitoring Committee

Role	Name	Contact details
Chair	Peter Sandercock	[redacted]
Members	Janet Darbyshire	[redacted]
	Robert Fowler	[redacted]
	David Lalloo	[redacted]
	David DeMets	[redacted]
	Mohammed Munavvar	[redacted]
	Adilia Warris	[redacted]
	Janet Wittes	[redacted]
Unblinded statisticians	Jonathan Emberson	[redacted]
	Natalie Staplin	[redacted]

b. Steering Committee

Role	Name	Address
Principal Investigators (blinded)	Peter Horby	[redacted]
	Martin Landray	[redacted]
Study Statisticians (blinded)	Enti Spata	[redacted]

RECOVERY Central Coordinating Office
Nuffield Department of Population Health (NDPH)
Richard Doll Building
University of Oxford
Old Road Campus
Roosevelt Drive
Oxford OX3 7LF
United Kingdom
recoverytrial@ndph.ox.ac.uk

(x)Signature log

I confirm that I have read and approved this Charter, and that I have no conflicts of interest (as described in Section 4.4 above). Only the Chair signature is required for minor changes to the DMC charter (e.g. updated contact details)

Name	Signature	Date
Peter Sandercock		
Janet Darbyshire		
Robert Fowler		
David Lalloo		
David DeMets		
Mohammed Munavvar		
Adilia Warris		
Janet Wittes		
